# Clinical validation and diagnostic accuracy of ^99m^Tc-EDDA/HYNIC-TOC compared to ^111^In-DTPA-octreotide in patients with neuroendocrine tumours: the LACOG 0214 study

**DOI:** 10.3332/ecancer.2023.1582

**Published:** 2023-07-26

**Authors:** Cristina M Moriguchi-Jeckel, Rafael R Madke, Graciane Radaelli, Alice Viana, Patrícia Nabinger, Bruna Fernandes, Gustavo Gössling, Eduardo H Berdichevski, Eduardo Vilas, Juliana Giacomazzi, Matheus Soares Rocha, João Alfredo Borges, Elias Hoffmann, Samuel Greggio, Gianina T Venturin, Carlos H Barrios, Facundo Zaffaroni, Gustavo Werutsky, Jaderson C da Costa

**Affiliations:** 1 Instituto do Cérebro do Rio Grande do Sul – Brain Institute (BraIns), Pontifícia Universidade Católica do Rio Grande do Sul (PUCRS), Av Ipiranga, Porto Alegre 90619-900, Brazil; 2 Escola de Ciências da Saúde e da Vida (PUCRS), Av Ipiranga, Porto Alegre 90619-900, Brazil; 3 Grupo RPH, Av Ipiranga, Porto Alegre 90619-900, Brazil; 4 Latin American Cooperative Oncology Group (LACOG), Av Ipiranga, Porto Alegre 90619-900, Brazil; 5 Hospital São Lucas da PUCRS, Av Ipiranga, Porto Alegre 90610-001, Brazil; 6 P3DMED, Rua Gomes Jardim, 201 Sala 1109A, Porto Alegre 90620-130, Brazil; 7 Núcleo de Imagens Médicas (Nimed), P96A do Tecnopuc – PUCRS, Porto Alegre, Brazil

**Keywords:** neuroendocrine tumours, technetium TC^99m^, indium In^111^, radionuclide imaging

## Abstract

^99m^Tc-EDDA/HYNIC-TOC is an easily available and cheaper radionuclide that could be used for somatostatin-receptor-based imaging of neuroendocrine tumours (NETs). We aimed to evaluate the diagnostic performance of ^99m^Tc-EDDA/HYNIC-TOC compared to^111^In-DTPA-octreotide in patients (pts) with NETs. We performed a prospective diagnostic study including pts with biopsy-confirmed NET and at least one visible lesion at conventional imaging. Two independent nuclear medicine physicians evaluated pts who underwent ^99m^Tc and ^111^In scans and images. The primary outcome was comparative diagnostic accuracy of ^99m^Tc and ^111^In. Secondary outcomes include safety.

Nine pts were included and performed 14 paired scans. Overall, 126 lesions were identified. ^99m^Tc demonstrated superior sensitivity both when all images were analysed (93.7, 95% CI 88.1% – 96.8% versus 74.8%, 95% CI 66.6 – 81.6%, *p* < 0.001) and when liver-specific images were analysed (97.8%, 95% CI 92.7% – 99.5% versus 85.1%, 95% CI 76.6% – 91.0%, *p* < 0.001). ^99m^Tc was also associated with a lower negative likelihood ratio (LR) (0.002, 95% CI 0.009 – 0.1 versus 0.19, 95% CI 0.12 – 0.42, *p* = 0.009) when evaluating hepatic lesions. Adverse events happened in 3 pts after ^111^In and in 2 pts after ^99m^Tc, all grade 1. The ^99m^Tc demonstrated a higher sensitivity overall and a better negative LR in liver-specific images compared to ^111^In in pts with NETs. Our findings suggest that ^99m^Tc is an alternative to ^111^In and is especially useful in ruling out liver metastases. NCT02691078.

## Introduction

Neuroendocrine tumours (NETs) are rare neoplasms that arise from epithelial cells with neuroendocrine features located primarily in the lungs, pancreas and gastrointestinal tissues [1, 2]. Clinical presentation and prognosis are extremely variable, from slow-growing well-differentiated disease to highly aggressive undifferentiated tumours [3]. The worldwide incidence is estimated to be around 50 cases per million inhabitants and has increased in the past decades, mostly due to better imaging [4–6].

Until the second decade of the 2000s, the reference product for scintigraphy examinations was ^111^In-DTPA-octreotide, as it was the only product registered worldwide, including Brazil. However, with the introduction of positron emission tomography (PET) scan with 68Ga-DOTA-octapeptides (DOTATATE, DOTATOC and DOTANOC), which provide a superior image pattern compared to those obtained by single photon emission computed tomography (SPECT), a new gold standard was established [7–12].

However, these methods have some drawbacks that affect their availability. In Brazil, ^111^In-DTPA-octreotide is supplied by a single institution (*IPEN – Instituto de Pesquisas Energéticas e Nucleares*), which is discontinuing its commercial production. Besides, it has high production costs, needs special cameras and collimators, and has long-term radioactivity that may harm patients and technicians [13]. While 68Ga-DOTA-octapeptides are still under regulatory approval by the Brazilian Health Agency (*ANVISA – Agência Nacional de Vigilância Sanitária*), the ^68^Ga labelling process requires a more costly and time-consuming process than the one with ^99m^Tc. Furthermore, in most low- and middle-income countries (LMICs), facilities and specialised professionals able to produce and distribute radiopharmaceuticals are lacking. Finally, SPECT cameras availability is still considerably higher than PET scans worldwide. Therefore, identifying other cheaper and quickly produced radiopharmaceuticals is currently a global unmet need.

In previous studies, ^99m^TC-EDDA/HYNIC-TOC (^99m^Tc marked octreotide) demonstrated similar efficacy to ^111^In-DTPA-octreotide (OCTREOSCAN^®^) in images from tumours that express somatostatin receptors (SSTRs), such as several types of NETs [14–18]. ^99m^TcEDDA/HYNIC-TOC has a higher affinity for SSTR2 and lower affinity for SSTR3 and SSTR5 [19]. Furthermore, ^99m^Tc is also easily available in nuclear medicine facilities worldwide without special technologies. Thus, we conducted the Latin American Cooperative Oncology Group (LACOG) 0214 study, which aimed to evaluate the performance of scintigraphy using ^99m^Tc-EDDA/HYNIC-TOC compared with ^111^In-DTPA-octreotide for the diagnosis and staging of patients with NETs.

## Materials and methods

### Study design and eligibility criteria

We planned a prospective diagnostic accuracy study in patients with biopsy-proven diagnoses of NETs from any location. The inclusion criteria were histological diagnosis of NETs in any stage, provided the patient had at least one visible lesion on computed tomography or magnetic resonance imaging. Patients should also have an indication of a ^111^In scan and be at least 18 years old. Patients should not have received somatostatin analogues in the month preceding the scan. An independent review board approved this research, and informed consent was obtained from all participants included in the study.

### Study procedures

Clinical, demographic and pathological data were collected at baseline. All patients underwent vital signs assessment and basic biochemistry labs before the procedures. Patients then initially underwent the scintigraphy using ^99m^Tc, followed by ^111^In scan in 2 days. Both scans were performed at the nuclear medicine facilities of the Brain Institute (BraIns). Two independent nuclear medicine physicians who were aware of the study evaluated all images.

The type of gamma camera used for the imaging process was the Forte Gamma Camera, manufactured by Philips. The matrix size was 64 × 6. SPECT/CT fusion imaging was conducted, allowing for enhanced anatomical localisation. The acquired images were analysed using the PEGASUS software. The Supplementary Information describes the protocol information.

Patients could undergo more than one pair of scans in case of re-evaluation after therapy initiation, as clinically indicated. Information was also collected regarding the number, location and intensity of lesions’ uptake.

The ^99m^Tc-EDDA/HYNIC-TOC images were obtained using a gamma camera with low energy collimators, focused on the photopeak of the ^99m^Tc (140.5 keV) with the symmetrical opening of 20% and injected activity of 10 mCi. We performed images of whole-body scans with SPECT 1 and 4 hours after radiopharmaceutical injection, in dorsal decubitus, with a 13 cm/minute velocity. The ^111^In-DTPA-octreotide images were performed in a gamma camera composed of collimators of medium energy, centred in the two photo peaks of the ^111^In (173 and 247 keV) with the symmetrical opening of 20% and activity injected of 6 mCi. Whole-body scan images were performed 4 and 24 hours after radiopharmaceutical injection, dorsal decubitus position, and 10 cm/minute velocity. SPECT was performed 24 hours after the injection of the radiopharmaceutical. Optional abdomen images were made after 48 hours of injection if bowel movements had interfered with image quality.

### Study endpoints

The primary outcome was the diagnostic accuracy of the ^99m^Tc-EDDA/HYNIC-TOC compared to ^111^In-DTPA-octreotide for diagnosis or staging of patients with NETs, irrespective of the primary site. Secondary outcomes included the number of NETs lesions visualised with radiopharmaceutical agents and safety.

Reports of acquisition-related medical complications evaluated adverse events and toxicity. Grading of adverse events was performed using the common terminology criteria for adverse events grading system.

### Statistical analysis

We estimated that 39 tests would have to be performed to demonstrate sensitivity and specificity of at least 90% with an acceptable error of 10% points for ^99m^TC compared to ^111^In, with a significance level of 95% and a 10% loss and refusal rate. Categorical variables were described using count and percentage, and numerical variables were summarised using mean and SD. Binary diagnostic tests’ properties were estimated and are presented with 95% Cis, including sensitivity, specificity, positive LR and negative LR. Positive and negative predictive values were intentionally not calculated once the sample was composed of patients with the disease on conventional imaging. Thus, prevalence and predictive values are not estimable from our sample. For all comparisons of sensitivities, specificities and LRs, global comparisons using the Wald test were performed initially and followed by individual comparisons with multiple comparisons correction using the Holm [20] method. Differences in sensitivities and specificities, LRs, and their 95% Cis were calculated using the Roldán-Nofuentes and Sidaty-Regad methods [21, 22]. Analysis was performed in *R*, version 4.0.5, using the Compbdt package [23]. This study was registered at clinical trials with the identifier NCT02691078 and is being reported according to the last version of the Standards for Reporting Diagnostic Accuracy Studies (STARD) guidelines [24–26] from the Equator Network.

## Results

Between May 2016 and July 2017, 14 scans were performed on 9 patients. Five patients performed both tests once, three patients twice, and one patient thrice. Unfortunately, the trial was stopped early due to under-enrolment. Patients were diagnosed from October 2007 to September 2016 and were examining disease re-evaluation after treatment. All nine patients were white, and five patients (55.6%) were female. The location of each patient’s primary tumour, baseline characteristics and comorbidities are described in Table 1.

Overall, 126 lesions were identified on the 14 scans. Two scans demonstrated an uncountable number of lesions, estimated at least 20. All 14 scans were positive using ^99m^Tc, while 4 out of the 14 scans were false negatives using ^111^In contingency tables presenting ^99m^TC and ^111^In scans’ performance according to image acquisition are presented in Table 2.

Estimated sensitivities, specificities and LRs are presented in Table 3. Overall, ^99m^Tc demonstrated higher sensitivity for identifying lesions in all images (*p* < 0.001, 95% CI for the difference 9.54% – 27.66%) and in liver-specific images (*p* < 0.001, 95% for the difference: 4.72% – 20.27%). However, the difference in sensitivities of ^99m^Tc and ^111^In when only whole-body images were considered was not statistically significant (*p* = 0.06). In addition, no statistically significant differences in specificity were found.

Regarding comparing LRs, the global Wald test demonstrated a statistically significant difference in LRs of ^99m^Tc and ^111^In when calculated for all images (*p* = 0.003) and liver-specific images (*p* = 0.025). However, after multiple comparison adjustments, only the difference of negative LR in liver-specific images was considered statistically significant (^99m^Tc: 0.002, 95% CI 0.009 – 0.1 versus ^111^In: 0.19, 95% CI 0.12 – 0.42, *p* = 0.009).

Adverse events were detected in three patients after ^111^In scans: pruritus [1], constipation [1], vomiting and diarrhoea [1], all grade 1. Two patients experienced adverse events after ^99m^Tc scans: pruritus [1] and vomiting and diarrhoea [1], all grade 1. No severe adverse events happened during the study.

**Figure 1.**
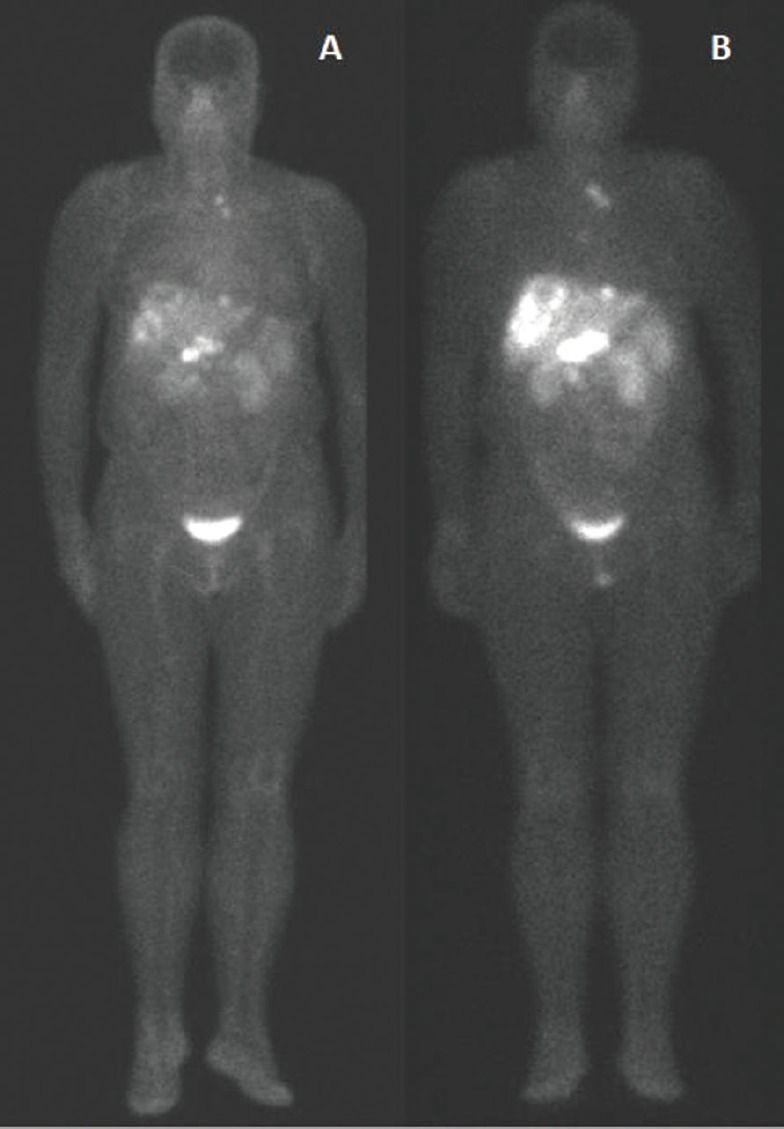
^99m^Tc-EDDA/HYNIC-TOC compared to ^111^In-DTPA-octreotide. Example of scintigraphy examination using (a): ^111^In-DTPA-octreotide and (b): ^99m^Tc-EDDA/HYNIC-TOC in the same patient, showing a more significant number of foci of uptake using ^99m^Tc.

In Figure 1, we have an example of the same patient evaluated by both imaging methods, showing a more significant number of foci of uptake with ^99m^Tc-EDDA/HYNIC-TOC compared to ^111^In-DTPA-octreotide.

## Discussion

The LACOG 0214 study was the first developed in Latin America on clinical validation of ^99m^Tc-EDDA/HYNIC-TOC scan for diagnosing and staging patients with NET. Our results suggest that ^99m^Tc, a cheaper and more available radiopharmaceutical, has an overall higher sensitivity than ^111^In, with better negative LR for liver metastasis in patients with NET.

Unfortunately, a lower-than-expected accrual resulted in early study termination and impacted our sample size. Causes for the under-enrolment cannot be ascertained. Consequently, our CI estimates for the differences in important parameters are comprehensive. For example, the 95% CI of the difference in sensitivity favouring ^99m^TC in all images is 9.54% – 27.66%, and in liver-specific images is 4.72% – 20.27%. However, we believe these imprecisions on the uncertainty are unfortunately inherent to the study of rare diseases such as NETs, which naturally imply smaller sample sizes and broader Cis. In this specific case, this limitation was addressed as conservatively as possible by calculating the 95% Cis with the Wald interval using the Bonett–Laplace adjustment, which was demonstrated to be superior to other methods in the previous studies [27].

Despite those limitations, the demonstration of a higher sensitivity overall, and especially a very low negative LR, of which the superior limit of the 95% CI is 0.1, supports that ^99m^Tc may be an adequate alternative to ^111^In. Furthermore, it provides a better capacity to rule out somatostatin analogue uptake in the presence of liver images consistent with metastases in patients with NETs. This is even more important considering that ^99m^Tc is associated with other beneficial characteristics, especially its availability.

Among the studies evaluating the ^99m^Tc, a study including 173 patients with NETs who underwent ^99m^Tc-EDDA/HYNIC-TOC scans as part of their clinical management found for pancreatic NETs a sensitivity of 94.6%, a specificity of 73.3%, and an accuracy of 90.1%. Gastrointestinal NETs found a sensitivity of 86.7%, specificity of 71.4%, and accuracy of 80.3% [28]. A study comparing ^99m^Tc-EDDA/HYNIC-TOC with ^111^In-DTPA-octreotide for diagnosis of SSTR-expressing tumours in 41 patients revealed a higher sensitivity of ^99m^Tc as compared with ^111^In as an imaging agent for the localisation of SSTR-expressing tumours [15].

Another study investigated 495 NETs patients and found an overall sensitivity of the ^99m^Tc-EDDA/HYNIC-TOC of 80%, specificity of 92%, positive predictive value of 98%, negative predictive value of 47%, and accuracy of 82% [29].

A systematic review analysing studies coupled two by two comparing the peptide-based radiopharmaceuticals for PET and SPECT, ^111^In-DTPA-Octreotide, ^99m^Tc-EDDA/HYNIC-TOC, ^68^Ga-DOTATATE/TOC and ^64^Cu-DOTATATE in the evaluation of NETs described true positivity rates of, respectively, 63.7%, 58.5%, 78.4% and 82.4% [30].

A literature review of ^99m^Tc highlighted its wider availability, low cost and long decay compared to peptides labelled with 68 Ga. It concluded that the ^99m^Tc could be proposed for a dosimetry evaluation of patients undergoing peptide receptor radionuclide therapy and for non-oncologic indications of radiolabelled somatostatin analogue (SSA) [8].

Our data reinforce that the diagnostic utility of the ^99m^Tc-EDDA/HYNIC-TOC gain is very likely limited compared to newer but less available technologies such as ^68^Ga-DOTATATE PET/CT. Still, clinical validation and diagnostic accuracy of ^99m^Tc and other markers are essential to LMICs, where the production and acquisition of radiopharmaceuticals are challenging [31, 32]. Thus, the possibility of using a more affordable and available radiopharmaceutical directly impacts care in these regions. Furthermore, even the demonstration of equivalent or non-inferior alternatives, considering other countries’ different legal constraints and regulations, impacts nuclear medicine services in LMICs due to their fragile supply chains [33]. For example, in Brazil, except for products with a half-life of fewer than 2 hours, production is a governmental monopoly, and the necessary resources are usually produced in small nuclear reactors that cannot have commercial radiopharmaceuticals. Thus, most Brazilian nuclear medicine services use foreign-produced radiopharmaceuticals and are dependent on external production, which leads to insecurity in the supply of radionuclides, as happened during the 2009 ^99^Mo ‘crisis’ [34, 35].

Nevertheless, despite PET-CT being an increasingly used imaging method, it is also unavailable for many patients outside high-income countries [36]. For those countries which cannot afford such health expenditure, ^99m^Tc use may be the most cost-effective strategy [33]. In the context of a rare disease, this gap may be even more significant since rare diseases such as NETs rarely have specifically designed guidance on healthcare systems and payers. Additionally, rare cancers are subject to natural limitations on clinical trial design and implementation, and this should be acknowledged when evaluating data for clinical use. Thus, a greater degree of uncertainty about therapies or diagnostic tests should also be accommodated for decision-making, as stated by the rare cancers Europe consensus panel [23].

## Conclusion

Despite its early termination, the LACOG 0214 trial demonstrated a superior sensitivity of ^99m^Tc-EDDA HYNIC-TOC compared to ^111^In-DTPA-octreotide in patients with NETs with a visible lesion on conventional imaging. The ^99m^Tc was also superior regarding its negative LR when evaluating hepatic lesions, suggesting that ^99m^Tc may be especially useful in ruling out liver metastases. Considering radiopharmaceutical acquisition is especially challenging for LMIC, the clinical validation of cheaper and more available molecules is a potential method to overcome this barrier in care.

## Conflicts of interest statement

Dr Moriguchi-Jeckel and Dr Werutsky report grants from Financiadora de Estudos e Projetos (FINEP) for the conduct of the study. Drs R. Madke, Viana, Nabinger, and Fernandes report employment relationships with the RPH Group. Dr. Werutsky reports personal fees from AstraZeneca, Bayer, Beigene, Daiichi Sankyo, Genentech/Roche, GSK, Lilly, MSD, Novartis, Pfizer, Sanofi, and Seattle Genetics outside the submitted work.

The other authors declare that they have no conflict of interest.

## Funding statement

This project obtained financial support from the Financier of Studies and Projects (FINEP), linked to the Ministry of Science, Technology and Innovation of Brazil, within the National Plan for Science and Technology framework. This project was also supported by the RPH Group, which provided the HYNIC-Octreotide used in this trial, and by the Latin American Cooperative Oncology Group (LACOG).

## Ethical statement

All procedures performed in studies involving human participants followed the ethical standards of the institutional and national research committee and with the 1964 Helsinki Declaration and its later amendments or comparable ethical standards.

## Author contribution

All authors contributed substantially to the project, as defined by the Committee of Medical Journal Editors, participating in the design of the study, the data interpretation, the article's writing, the critical review of the intellectual content, and the final approval of the version to be published.

## Figures and Tables

**Table 1.** Study subjects' characteristics.

Characteristics	Patients (*n* = 9)
Age (years)	60 (±12)
Ethnicity	
White	9 (100%)
Sex	
Male	4 (44.4%)
Female	5 (55.6%)
Comorbidities	
Hypertension	4 (44.4%)
Diabetes	3 (33.3%)
COPD	1 (11.1%)
Primary tumour	
Liver	6 (66.7%)
Small intestine	3 (33.3%)
Menopausal status (*n* = 5)	
Post-menopausal	5 (100%)
Hemoglobin (g/dL)	13.7 (±1.8)
Creatinine (mg/dL)	1.2 (±0.4)
Weight (kg)	75.4 (±18.96)
Height (m)	1.63 (±0.05)

Data are presented as mean (±SD) or *n* (%). COPD: chronic obstructive pulmonary disease

**Table 2.** ^99m^TC and ^111^In scans’ performance according to image acquisition.

	^99m^Tc scan	^111^In scan		Total
		Positive	Negative	
A – Any images	Positive	88	7	95
	Negative	31	0	31
	Total	119	7	126
B – Liver-specific images	Positive	79	13	92
	Negative	0	5	5
	Total	79	18	97
C – Whole-body images	Positive	9	18	27
	Negative	7	4	11
	Total	16	22	38

Contingence table of ^99m^TC and ^111^In scans’ performance on detection of lesions in (A) Any image, (B) Liver-specific images, or (C) Whole-body images

**Table 3.** Performance parameters of ^99m^Tc-EDDA/HYNIC-TOC and ^111^In-DTPA-OCTREOTIDE.

	(A) Any image			(B) Liver-specific images			(C) Whole-body images		
	^99m^Tc	^111^In	*p*	^99m^Tc	^111^In	*p*	^99m^Tc	^111^In	*p*
Sensitivity	93.7% (88.1 – 96.8)	74.8% (66.6 – 81.6)	< 0.001	97.8% (92.7 – 99.5)	85.1% (76.6 – 91.0)	< 0.001	77.1 (61.1 – 88.1)	45.7% (30.4 – 61.7)	NS*
Specificity	50% (15.0 – 84.9)	50% (15.0 – 84.9)	0.61	75% (41.5 – 93.4)	75% (41.5 – 93.4)	0.61	71.4 (36.4 – 92.3)	71.4% (36.4 – 92.3)	
Positive LR	1.49 (0.88 – 4.52)	1.87 (1.11 – 5.64)	0.75	3.91 (1.61 – 10.90)	3.40 (1.4 – 10.17)	0.84	2.7 (1.13 – 8.52)	1.6 (0.62 – 5.56)	NS*
Negative LR	0.50 (0.28 – 1.55)	0.12 (0.05 – 0.42)	0.08	0.02 (0.009 – 0.1)	0.19 (0.12 – 0.42)	0.009	0.32 (0.16 – 0.78)	0.76 (0.52 – 1.61)	

* When the global Wald test was not significant, individual comparisons were not performed and thus are not presented

^99m^Tc: ^99m^Tc -EDDA/HYNIC-TOC; ^111^In: ^111^In -DTPA-OCTREOTIDE; LR: Likelihood Ratio; PV: Predictive Value; NS: Not Significant

Estimated sensitivities, specificities, and LRs of ^99m^Tc-EDDA/HYNIC-TOC and ^111^In-DTPA-OCTREOTIDE according to disease detection in (A) Any images, (B) Liver-specific images and (C) Whole-body images

## Supplementary information

### Study protocol

#### Preparation of the patient for the exam for the two radiopharmaceuticals:

The patient must fast for 4 hours for solids before administration of the radiopharmaceutical, and must remain well hydrated before and after the examination, in order to reduce the radiation dose. The use of non-radiolabeled somatostatin analogues may reduce the sensitivity of the method. Therefore, the exams will be carried out just before the administration of long-acting formulas. Short-acting formulas will be suspended 24 hours before the exam.

### Instrumentation

#### Radiopharmaceutical: HYNICTOC-EDDA-99mTc

The images will be performed in the same gamma-camera; with low-energy collimators, centred on the Tc-99m photopeak (140.5 keV) with a symmetrical aperture of 20%. The injected activity will be 10 mCi.

**Image acquisition protocol:**

- Scanning images of the entire body will be performed 1 and 4 hours after the injection of the radiopharmaceutical, in dorsal decubitus, with arms down, with a 1,024 × 512 matrix, at a speed of 13 cm/minute.
- Tomographic images (SPECT) of the areas of interest will be taken 4 hours after the injection of the radiopharmaceutical in dorsal decubitus, with a 64 × 64 matrix, 64 projections of 30 seconds and zoom of 1.

#### Radiopharmaceutical: OCTREOTIDEO – DTPA – In-111

The images will be taken in a gamma-camera composed of medium-energy collimators, centred on the two In-111 photopeaks (173 and 247 keV) with a symmetrical aperture of 20%. The injected activity will be 6 mCi.

**Image acquisition protocol:**

- Scanning images of the entire body will be performed 4 and 24 hours after the injection of the radiopharmaceutical, in dorsal decubitus, with arms down, matrix 1,024 × 512 and speed of 10 cm/minute.
- Tomographic images (SPECT) of the areas of interest will be taken 24 hours after the injection of the radiopharmaceutical in the supine position, with a 64 × 64 matrix, 64 projections of 35 seconds and a zoom of 1.
- Planar images of the abdomen, of 48 hours, are optional, in case there is interference of intestinal activity making it difficult to interpret the 24 hours images. In this case, they will be performed with 500,000 counts and a 128 × 128 matrix.
